# Obesity Disrupts CtBP2‐Mediated Maintenance of Transcriptional Equilibrium in Hypothalamic Feeding Circuitry

**DOI:** 10.1096/fj.202602322R

**Published:** 2026-07-29

**Authors:** Wanpei Chen, Kenta Kainoh, Kenji Saito, Takaaki Matsuda, Daichi Yamazaki, Yuto Kobari, Ayumi Nakata, Nao Aono‐Soma, Takafumi Miyamoto, Yuki Murayama, Yoko Sugano, Yoshinori Osaki, Hitoshi Iwasaki, Takashi Matsuzaka, Hitoshi Shimano, Motohiro Sekiya

**Affiliations:** ^1^ Department of Endocrinology and Metabolism, Institute of Medicine University of Tsukuba Ibaraki Japan; ^2^ Center for Cyber Medicine Research University of Tsukuba Ibaraki Japan; ^3^ Transborder Medical Research Center University of Tsukuba Ibaraki Japan

**Keywords:** AgRP, CtBP2, hypothalamus, metabolite sensor, NPY, obesity

## Abstract

Feeding behavior, which is crucial for all mammals, is regulated by a delicate equilibrium between the orexigenic and anorexigenic activities of hypothalamic neurons. This exquisite control of the neuropeptides that govern the feeding behavior could be a focal point in the pathogenesis of obesity. We reported that inactivation of C‐terminal binding protein 2 (CtBP2), a transcriptional corepressor with metabolite‐sensing capabilities, contributes to the pathogenesis of obesity in liver tissues and pancreatic β‐cells. Here, we describe a transcriptional system regulated by CtBP2 in the hypothalamus. Our global mapping of CtBP2 binding sites using ChIP‐seq combined with functional analyses revealed that CtBP2 functions as a corepressor for orexigenic neuropeptide promoters. In response to the metabolic abnormalities associated with obesity, CtBP2 undergoes allosteric inactivation and dissociates from these promoters, thereby resulting in the derepression of orexigenic neuropeptide expression. Consistently, the loss of CtBP2 in hypothalamic neurons in mice increases the expression of orexigenic neuropeptides, thus leading to increased feeding behavior. These findings highlight how obesity disrupts homeostatic mechanisms that normally maintain body weight within a healthy range.

## Graphical abstract

CtBP2, a transcriptional corepressor with metabolite sensing capabilities, maintains feeding homeostasis by preventing excessive food intake in the hypothalamic feeding center. CtBP2 limits food intake by inhibiting KLF4 within AgRP neurons and by promoting the inhibitory influence of POMC neurons on AgRP neurons. In obesity, however, obesity‐associated metabolic changes compromise the function of CtBP2, resulting in increased food intake. The mechanism may help explain, at least, the molecular basis underlying the tendency of individuals with obesity to overeat.

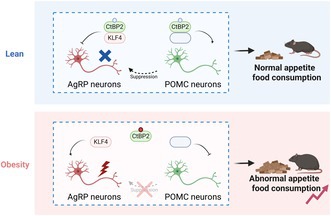

## 1 Introduction

Feeding behavior is essential for the viability and vitality of all mammals, and perturbations in this regulatory system, manifesting as hyperphagia or hypophagia, can lead to pathological conditions such as obesity or anorexia/cachexia [1]. Under normal conditions, robust homeostatic mechanisms, including the leptin‐mediated feedback loop [2], maintain body weight within a specific range. However, in obesity, these mechanisms are disrupted, allowing for the maintenance of elevated body weight. This raises the question of how obese individuals maintain their increased set point for body weight, and answering this question could allow us to overcome obesity.

The feeding center is anatomically located in the hypothalamus where two distinct neural populations expressing counterbalancing neuropeptides, orexigenic agouti‐related protein (AgRP)/neuropeptide Y (NPY) and anorexigenic pro‐opiomelanocortin (POMC)/cocaine‐amphetamine‐regulated transcript (CART, encoded by the *Cartpt* gene), play a pivotal role in maintaining optimal dietary intake [1]. AgRP/NPY neurons are activated during energy deprivation, and the intracerebroventricular administration of AgRP and NPY increases food intake [3, 4], although the relative dependency of the drive to seek food on these neurons remains controversial [5, 6, 7, 8, 9, 10]. Conversely, POMC is posttranslationally cleaved into bioactive peptides that primarily suppress appetite through melanocortin signaling [11], with some exceptions [12]. Another anorexigenic peptide, CART, is also produced via cleavage of its precursor form [13], adding redundancy and complexity to this anorexigenic system.

Feeding behavior is regulated by multiple systems, including hormonal regulation, neural network formation, and neurotransmitter uptake and release [14]. This redundancy is crucial given the essential nature of feeding behavior for the survival of all mammals. Transcriptional regulation also serves as one of the pivotal regulatory systems [15]. Central insulin signaling and leptin signaling, in part, activate phosphatidylinositol 3‐kinase (PI3K), resulting in the cytoplasmic sequestration of Forkhead box O1 (FoxO1). Since FoxO1 transactivates *Agrp* and *Npy* gene expression, insulin and leptin suppress food intake [16, 17]. Signal transducer and activator of transcription 3 (STAT3) is another transcription factor that has been extensively studied. Leptin signaling activates STAT3 to suppress *Agrp* and *Npy* gene expression and activates *Pomc* gene expression, resulting in its potent anorexigenic effects [17, 18]. Despite several technical challenges, other transcription factors, transcriptional cofactors, and epigenetic modifiers have also been shown to be involved in these central transcriptional regulatory systems [19, 20, 21]. Since some of them are shared across tissues, identifying pharmacologically targetable molecules with widespread distribution may lead to the development of attractive therapeutics with global benefits. In addition, given the close link between feeding and nutrition, transcriptional mechanisms coupling nutrient metabolism to feeding behavior may involve previously unrecognized regulatory factors.

C‐terminal binding protein (CtBP) transcriptional corepressors regulate gene expression by recruiting epigenetic regulators across various tissues and cells [22]. Since CtBPs lack DNA‐binding capability, they bind to specific transcription factors to access genomic regions. There are two isoforms in mammals, CtBP1 and CtBP2, with CtBP2 specializing in transcriptional regulation due to its nuclear localization signal in the N‐terminal region [23]. CtBP2 is notable for its metabolite‐sensing capabilities. It has a structural pocket called the Rossmann fold to accommodate metabolites. Upon binding to the pyridine dinucleotide metabolite nicotinamide adenine dinucleotide (NADH/NAD^+^), CtBP2 adopts a multimeric configuration, promoting interactions with transcription factors in most cases [24, 25]. Intriguingly, it was reported that CtBP2 can also respond to the redox state or glycolytic activity through its preferential binding affinity for NADH over NAD^+^ [24], albeit with some debates [26, 27]. We recently demonstrated that CtBP2 dimerization is inhibited upon binding to fatty acyl‐CoAs [28], which accumulate in the tissues of obese individuals. In healthy subjects, CtBP2 suppresses hepatic gluconeogenesis and lipogenesis, but this suppression is disrupted by fatty acyl‐CoAs in obesity, leading to diabetes and hepatic steatosis [28]. Additionally, CtBP2 monomerization impairs fatty acid oxidation in the livers of obese mice [29]. The role of CtBP2 in obesity extends beyond the liver, as the progressive decline of pancreatic β‐cell functions in obesity may also be due to obesity‐induced loss of CtBP2 [30]. The activation of CtBP2 provides protection against oxidative stress [31]. Thus, the metabolic inactivation of CtBP2 may underlie the pathogenesis of obesity across various tissues, whereas CtBP2 activation could be a promising therapeutic approach [32]. Recently, we further demonstrated that activated CtBP2 exerts geroprotective effects through a distinct mechanism [33].

In this study, we demonstrate that CtBP2 regulates the expression of key genes in the hypothalamic feeding center. CtBP2 serves as a corepressor of orexigenic neuropeptide expression. Obesity‐induced metabolic abnormalities inactivate CtBP2, and mice with genetic CtBP2 deletion, specifically in AgRP or POMC neurons, exhibit both increased expression of orexigenic genes and hyperphagia. These findings reveal a consistent and compelling relationship between CtBP2 and obesity, extending to central feeding regulation, and underscore the multifaceted role of CtBP2 in metabolic homeostasis.

## 2 Materials and Methods

### 2.1 Animals

The research protocol received approval from the Animal Care Committee of the University of Tsukuba (approval number 25‐113), and all experimental procedures involving animals were performed in accordance with the committee's guidelines. All of the mice utilized in this study were male and were maintained on a cycle consisting of 14‐h light and 10‐h dark, with unrestricted access to water and a standard chow diet. Zeitgeber time (ZT) was defined as ZT0 at lights on (05:00) and ZT14 at lights off (19:00).

We generated two cell type‐specific CtBP2‐deficient mouse models by crossing CtBP2 flox mice [28] with either *Agrp* promoter‐Cre transgenic mice (Jackson #012899) [34] (ACKO) or *Pomc* promoter‐Cre transgenic mice (Jackson #005965) (PCKO) [35]. CtBP2 flox/flox;Cre^+^ and CtBP2 flox/flox;Cre^−^ mice were intercrossed to generate experimental cohorts, and littermate controls were used in all experiments. Mice were maintained on the same genetic background and housed together to minimize environmental variability.

For fasting experiments, mice were fasted starting at ZT14 and sacrificed after 16 h of fasting (ZT7). For refeeding experiments, mice were fasted for 16 h starting at ZT14, followed by 1 h of refeeding beginning at ZT7, and sacrificed at ZT8. Under normal chow conditions, mice were sacrificed at ZT7. All sacrifices were performed at defined time points to minimize circadian variation. Hormone levels were assessed with the Milliplex Mouse Metabolic Hormone Expanded Panel (Merck). Daily food intake was measured in individually housed mice at ZT3 and ZT14. Food consumption was calculated by subtracting the weight of the remaining standard chow pellets from the preweighed amount that was provided. The mice were acclimated to individual housing for 7 days prior to the start of the recording period, during which time food intake was monitored for up to 21 consecutive days. Body weight was recorded once per week. Following a 12‐h overnight fast, the mice were refed with standard chow at ZT0, and both food intake and body weight were measured at 0, 14, 24, and 48 h after the onset of refeeding. Energy expenditure was determined using an ARCO‐2000 Mass Spectrometer System (Arcosystem Inc., Chiba, Japan).

### 2.2 Immunofluorescence

The mice were anesthetized and perfused with cold phosphate‐buffered saline (PBS), followed by 4% paraformaldehyde (Nacalai, 09154‐85, Japan). The brains were postfixed overnight at 4°C in 4% paraformaldehyde, and followed by 30% sucrose in PBS for 48 h at 4°C. The tissues were embedded in optimal cutting temperature compound (OCT; Sakura, 4583, Japan) and sectioned at a thickness of 40 μm using a cryostat (Leica, CM1860, Germany). Coronal sections corresponding to bregma −1.22 to −2.30 mm containing the hypothalamic arcuate nucleus. The sections were subsequently washed with PBS and blocked in 5% bovine serum albumin (BSA) and 0.1% Triton X‐100 in PBS for 30 min at room temperature. The sections were then incubated with primary antibodies, including mouse anti‐CtBP2 antibody (1:50; BD Biosciences, 612044, USA), mouse anti‐AgRP antibody (1:50; Santa Cruz, sc‐518077, USA), and rabbit anti‐POMC antibody (1:50; Cell signaling Technology, 23499, USA) at 4°C for 24 h. After being washed with PBS three times, the sections were incubated with anti‐mouse IgG Alexa 488 (1:500; Cell signaling Technology, USA) and anti‐rabbit IgG Alexa 555 (1:500, Cell signaling, USA) for 90 min at room temperature, after which the sections were counterstained with DAPI (1:1000; Dojindo, 340‐07971, Japan) for 5 min. The sections were visualized using a fluorescence microscope (BZ‐X710, Keyence, Japan). Image analysis was performed using the accompanying software (BZ Analyzer, version 3.6.0.0, Keyence).

### 2.3 Cell Culture and Luciferase Reporter Assay

The mHypoE‐N41 immortalized mouse hypothalamic cell line was purchased from CELLutions BIOSYSTEMS and cultured in Dulbecco's modified Eagle's medium (DMEM; Gibco 11 965) supplemented with 25 mM glucose, 100 U/mL penicillin and 100 μg/mL streptomycin sulfate, supplemented with 10% fetal bovine serum.

Human KLF4 expression plasmid was obtained from Addgene (#34593). The mouse promoter regions of *Npy* gene (−701 to +888), *Agrp* gene (−502 to −28), *Pomc* gene (−769 to −244), and *Cartpt* gene (−528 to −45) were PCR‐amplified and cloned into the PGL3 basic plasmid to construct luciferase reporter plasmids (Promega). These luciferase reporter plasmids and the pSV40‐Renilla plasmid were cotransfected into cells with either CtBP2 [28] and/or the KLF4 expression plasmids using Lipofectamine LTX reagent (Thermo) for 48 h. Luciferase activity was measured using the Dual‐Luciferase Reporter Assay System (Promega) on a Varioskan multimode microplate reader (Thermo), with values normalized to Renilla luciferase activity.

The siKLF4 (SASI_Mm01_00104983) and a non‐targeting control siRNA (SIC001) were purchased from Merck. Those siRNAs were transfected using Lipofectamine RNAiMAX Transfection Reagent (Invitrogen, 13 778 075) according to the manufacturer's instructions for 72 h.

### 2.4 Quantitative Real‐Time RT‐PCR

Total RNA was extracted using Sepazol Reagent (Nacalai) and cDNA was synthesized with PrimeScript RT Master Mix (Takara Bio). Quantitative real‐time PCR was conducted using SYBR Green on a 7300 Real‐Time PCR System (Applied Biosystems), with the data being normalized to the acidic ribosomal phosphoprotein P0 (*Rplp0*, 36B4).

### 2.5 RNA‐Seq Analysis and Downstream Data Mining

Samples were collected from ACKO mice under ad libitum feeding conditions and from PCKO mice following 16‐h fasting. The sequencing library was constructed with the NEBNext Ultra II Directional RNA Library Prep Kit for Illumina (New England Biolabs), and sequencing was performed on a NovaSeq 6000 system (Illumina). Subsequent procedures, including quality checks of the raw paired‐end reads, trimming, and alignment to the mm10 mouse reference genome, were performed as previously described [30]. Raw read counts were normalized to transcripts per million (TPM), and downstream pathway analyses were conducted using integrated Differential Expression and Pathway analysis (iDEP) software version 2.01 [36]. Data are available under GEO accession Nos. GSE266965 (ACKO) and GSE266966 (PCKO).

### 2.6 Chromatin Immunoprecipitation (ChIP)

Mice with diet‐induced obesity were fed a high‐fat diet (D12492, Research Diets) for 14 weeks beginning at 4 weeks of age. Leptin‐deficient *ob/ob* mice, aged 8 weeks, were procured from Oriental Yeast Co. Ltd. Hypothalamic tissues were harvested from these obese mice and their respective controls, which were fed ad libitum. The tissues were subsequently washed and fixed in 1% formaldehyde for 10 min at room temperature, after which cross‐linking was terminated by the addition of glycine to a final concentration of 125 mM. ChIP was subsequently performed using the Magna ChIP HiSens Chromatin Immunoprecipitation System (EMD Millipore). Chromatin fragmentation was achieved through micrococcal nuclease digestion (Cell Signaling), followed by sonication (Bronson Sonifier 250). The chromatin was then immunoprecipitated using either control IgG (Cell Signaling) or anti‐CtBP2 antibody (Active Motif, 61261). The quantification of immunoprecipitated and input DNA was conducted via real‐time PCR using primers specific to the *Npy* gene promoter. The following primer sequences were utilized: *Npy* forward, 5′‐aagtggctgtgggagtcacc‐3′; *Npy* reverse, 5′‐ctgtgagagaagagatccaccggt‐3′. The primers used for the negative control region are described in a previous study [28].

### 2.7 Re‐ChIP

HEK293 cells were transfected with HA‐KLF4 and CtBP2 expression plasmids. Chromatin was cross‐linked with 1% formaldehyde and quenched with glycine, fragmented by micrococcal nuclease digestion (Cell Signaling) followed by sonication (Bronson Sonifier 250), and subjected to a first immunoprecipitation using anti‐HA magnetic beads. Bound chromatin was eluted with HA peptide under non‐denaturing conditions and divided equally for a second immunoprecipitation using either control IgG (Cell Signaling) or anti‐CtBP2 antibody (Active Motif, 61261) with the Magna ChIP HiSens Chromatin Immunoprecipitation System (EMD Millipore) as described above. Immunoprecipitated DNA and input DNA were reverse cross‐linked and analyzed by quantitative PCR using primers targeting the human *NPY* gene promoter. *NPY* forward, 5′‐cggcgaggaagctccataa‐3′; *NPY* reverse, 5′‐aagtactatgctgtcgggcg‐3′.

### 2.8 ChIP‐Seq Analysis and Downstream Data Mining

Chromatin immunoprecipitated DNA using the anti‐CtBP2 antibody (Active Motif 61261) and input DNA were obtained from normal mice fed a chow diet ad libitum. The sequencing library was prepared with NEBNext Ultra II DNA Library Prep Kit (New England Biolabs), and paired‐end sequencing was conducted on an Illumina NovaSeq 6000 System. Following the trimming of the low‐quality bases and adapter sequences with Trimmomatic software (version 0.38), the reads were aligned to the mouse reference genome mm10 using Bowtie2 (version 2.3.4.2), resulting in 33 743 782 final reads. Binding peaks were identified using model‐based ChIP‐seq analysis (MACS2, version 2.1.2). A signal heatmap was generated with the computeMatrix and plotHeatmap tools in deepTools. The abundance of uniquely mapped reads across genomic features was quantified using feature counts (version 1.6.3). Consensus motifs of CtBP2 binding sites were delineated using the Multiple EM for Motif Elicitation (MEME)‐ChIP program. To identify candidate transcription factors that bind to CtBP2, these motifs were compared against databases of known motifs using TOMTOM. Data are available under GEO accession No. GSE266964.

### 2.9 Indirect Quantification of Fatty Acyl‐CoAs

Fatty acyl‐CoAs were measured as previously reported [28]. Total lipids were extracted from hypothalamic tissue using a methanol:chloroform:water mixture (2:1:0.8, v/v/v), and 500 μL of the supernatant was collected and air‐dried. Dried lipid extracts were re‐suspended in water to solubilize fatty acyl‐CoAs. Acyl‐CoA oxidase (Wako, W01A31T‐17, Japan) was used to enzymatically oxidize fatty acyl‐CoA substrates to trans‐2‐enoyl‐CoA with concomitant production of hydrogen peroxide.

After acyl‐CoA oxidase treatment, hydrogen peroxide generated in the reaction was quantified using the Hydrogen Peroxide Fluorometric Detection Kit (Enzo, ADI‐907‐028, USA) according to the manufacturer's instructions. Briefly, reaction buffer, detection reagent, horseradish peroxidase, and 50 μL of sample or standard were mixed in a black 96‐well microplate, and fluorescence (λ595 ex/λ590 ex) was measured using a plate reader.

To translate hydrogen peroxide signals to fatty acyl‐CoA equivalents, a parallel calibration curve was constructed by subjecting known concentrations of oleoyl‐CoA (0–40 μM) to the same acyl‐CoA oxidase treatment and subsequent hydrogen peroxide measurement. Final fatty acyl‐CoA levels were expressed as oleoyl‐CoA equivalents per mg tissue after normalization.

### 2.10 Immunoprecipitation and Western Blot Analysis

Proteins were extracted from tissues using buffer A (50 mM Tris–HCl pH 7.4, 150 mM NaCl, 1% Nonidet P‐40, 1 mM EDTA, 10 mM NaF, and 2 mM Na_3_VO_4_) supplemented with complete protease inhibitors (Sigma‐Aldrich) and subjected to SDS‐polyacrylamide gel electrophoresis.

For HA‐tag co‐immunoprecipitation, anti‐HA magnetic beads (Thermo, 88836) were used to pull down HA‐KLF4 from lysates prepared in Buffer A containing 1% NP‐40 and protease inhibitors. After incubation for 2 h at 4°C with rotation, beads were washed four times with the same buffer and eluted with HA peptide.

Nuclear extracts were prepared using the NE‐PER Nuclear and Cytoplasmic Extraction System (Thermo, 78835) according to the manufacturer's instructions. Equal amounts of nuclear protein were subjected to immunoprecipitation using anti‐HA magnetic beads as described above. Lamin A/C (Proteintech, 10 298‐1‐AP) was used as a nuclear fraction marker where indicated.

For endogenous co‐immunoprecipitation from mouse hypothalamus, anti‐CtBP2 antibody (BD, 612044) or control mouse IgG was cross‐linked to Dynabeads Protein G (Invitrogen, DB10004) with dimethylpimelimidate (Sigma, D8388) [28]. Hypothalamic tissues from HFD‐fed mice or controls (pooled from three mice per group) were lysed in Buffer A containing 1% NP‐40 and protease inhibitors, and clarified lysates were incubated with antibody‐conjugated beads for 4 h at 4°C. Beads were washed four times with lysis buffer, eluted in SDS sample buffer, and analyzed by western blotting.

The membranes were incubated with anti‐CtBP2 (BD, 612044) and anti‐alpha tubulin (Sigma, T6199). Secondary antibodies conjugated with horseradish peroxidase (Cell Signaling Technology) were used for detection, and signals were visualized using an enhanced chemiluminescence system (Bio‐Rad).

### 2.11 Quantification and Statistical Analysis

Statistical analyses were performed using a two‐tailed unpaired Student's *t*‐test or two‐way ANOVA followed by Tukey's multiple comparisons test.

## 3 Results

### 3.1 CtBP2 Orchestrates Transcription of a Wide Range of Genes in the Hypothalamus and Serves as a Repressor of Orexigenic Neuropeptide Expression

**FIGURE 1:**
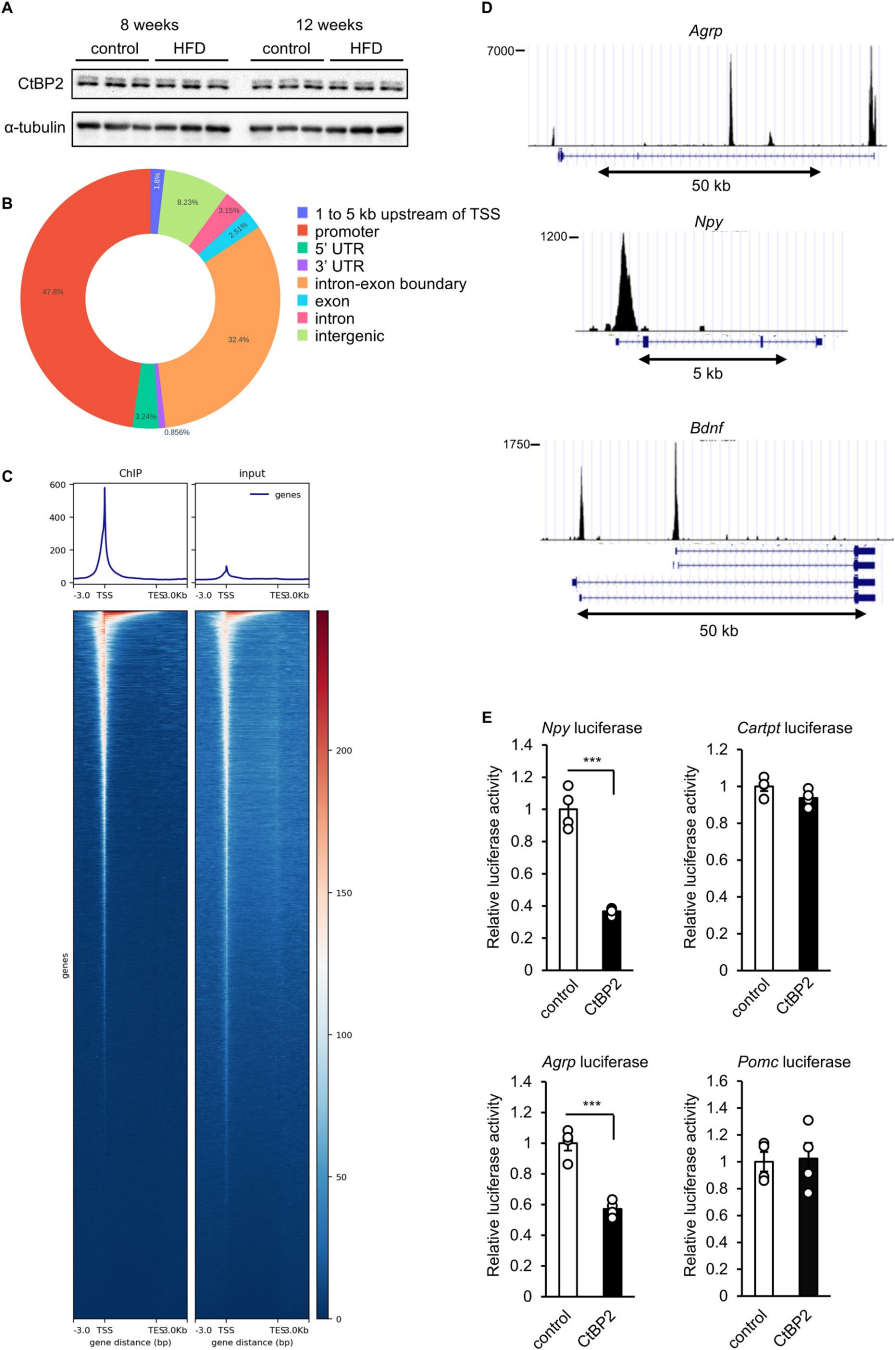
CtBP2 serves as a transcriptional repressor of orexigenic genes in hypothalamic neurons. (A) Protein expression of CtBP2 in hypothalamic tissues obtained from diet‐induced obese mice (fed a high‐fat diet (HFD) for 8 weeks or 12 weeks) and their controls. (B) Percentage distribution of CtBP2 ChIP‐seq peaks with respect to gene features in normal hypothalamic tissue. (C) Distribution of CtBP2 ChIP‐seq peaks and input control signals relative to the TSSs. (D) Genome‐wide mapping of CtBP2 binding regions in the hypothalamus of normal mice: A ChIP‐seq analysis. CtBP2 was recruited to the promoters of orexigenic genes and genes important for neuronal functions, as exemplified by *Bdnf*. (E) Promoter activities of the *Npy*, *Cartpt, Agrp* and *Pomc* genes. The exogenous expression of CtBP2 reduced *Npy* and *Agrp* promoter activities, whereas *Cartpt* and *Pomc* promoter activities remained unchanged in mHypoE‐N41 hypothalamic cells (*n* = 4). The data are expressed as the mean ± SEM with individual data points. ***p < 0.001, as determined by the Student's *t*‐test.

A series of previous observations suggest the involvement of CtBP2 in the pathogenesis of obesity across tissues [32], and in addition, a recent human study reported an association between anorexia and single nucleotide polymorphisms in CtBP2 gene loci [37]. Thus, we investigated the potential role of CtBP2 in the hypothalamic feeding center. Consistent with its ubiquitous expression, CtBP2 protein was detected in hypothalamic tissues in mice (Figure 1A). We then globally mapped CtBP2 binding sites using chromatin immunoprecipitation followed by high‐throughput sequencing (ChIP‐seq) to gain comprehensive insights into the repertoire of genes regulated by CtBP2 in this tissue. Analysis of the peak distribution relative to genomic features revealed that CtBP2 was frequently recruited to transcriptional start sites (TSSs), with peaks also being mapped to other genomic features, indicating its role in transcriptional regulation in this tissue (Figure 1B,C). We observed enrichment of peaks around promoter regions of genes encoding orexigenic neuropeptides and key genes regulating neuronal functions, such as brain‐derived neurotrophic factor (*Bdnf*) (Figure 1D). Although small peaks for anorexigenic neuropeptides were observed, they were not robust, suggesting that CtBP2 may preferentially regulate orexigenic neuropeptide expression (Figure S1A). We further surveyed other orexigenic neuropeptides involved in the feeding regulation (Figure S1B). While CtBP2 was modestly recruited to the galanin (*Gal*) promoter, we did not observe any peaks at the orexin (*Hcrt*) and MCH (melanin‐concentrating hormone, *Pmch*) promoters. Despite the absence of CtBP2 at the promoters of orexin and MCH, CtBP2 recruitment was detected at the promoters of receptors for these orexigenic neuropeptides. Intriguingly, CtBP2 was selectively recruited to the promoter of type 1 orexin receptor (*Hcrtr1*) among the two orexin receptors (Figure S1B). In support of this notion, the exogenous expression of CtBP2 in an immortalized mouse hypothalamic cell line (mHypoE‐N41) repressed the promoter activities of the *Npy* and *Agrp* genes, whereas *Pomc* and *Cartpt* gene promoter activities were unaffected (Figure 1E).

**FIGURE 2:**
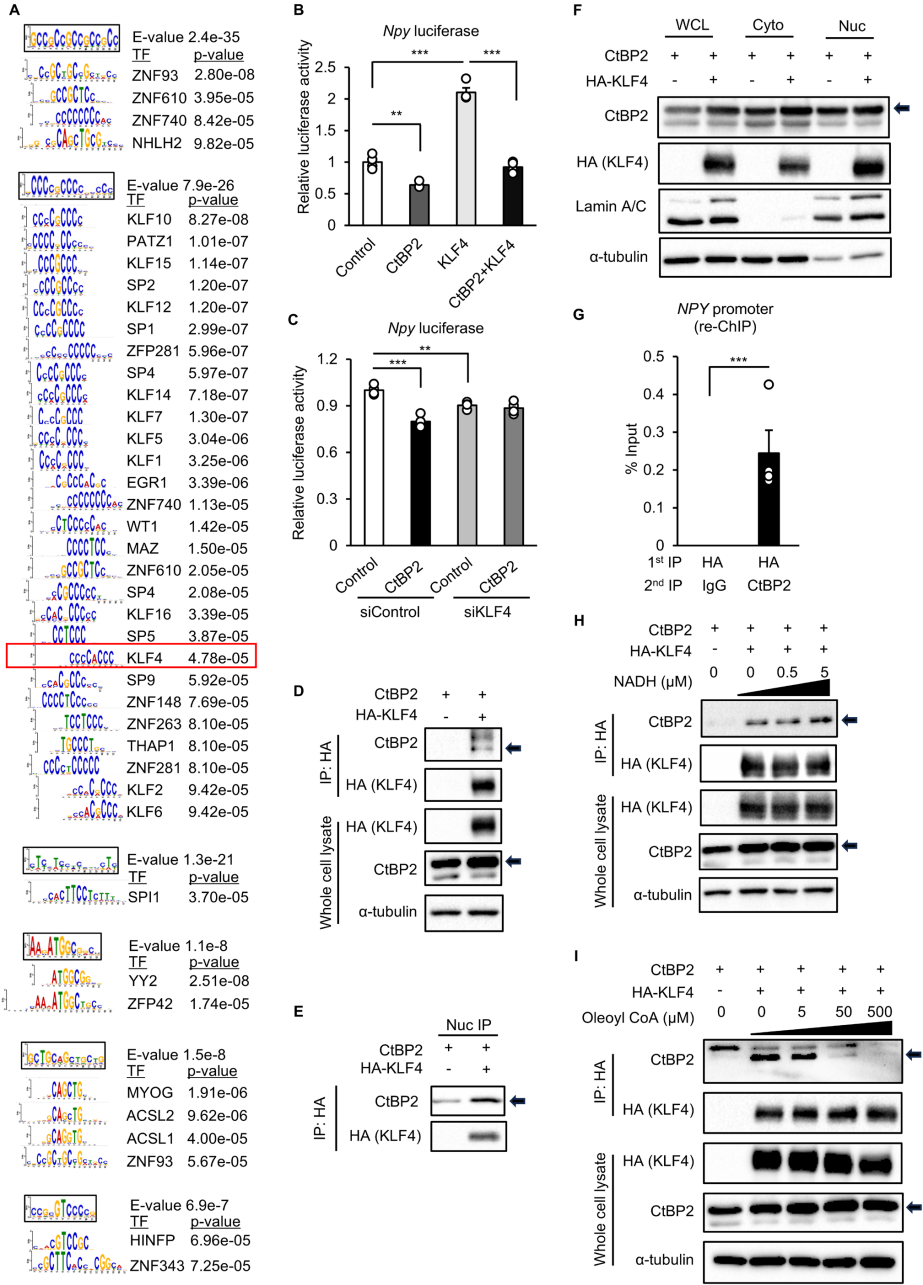
Integration of motif and gene ontology analyses identifies CtBP2/KLF4 transcriptional complex. (A) The motifs enclosed by rectangles are CtBP2‐binding motifs enriched in the ChIP‐seq data. E values for each motif are indicated. Predictions of known transcription factors targeted by CtBP2 based on sequence similarity are shown below along with the *p* values. The KLF4‐biding motif is highlighted by a red rectangle. (B) Alterations in *Npy* promoter activities induced by exogenous expression of CtBP2 and/or KLF4 in mHypoE‐N41 (*n* = 4). (C) Promoter activities of the *Npy* gene in mHypoE‐N41 hypothalamic cells in the presence or absence of KLF4 knockdown and/or CtBP2 overexpression (*n* = 3). (D) The interaction of CtBP2 with KLF4 was detected by co‐immunoprecipitation in HEK293 cells with exogenous expression of CtBP2 and/or KLF4. (E, F) Co‐immunoprecipitation of HA‐KLF4 and CtBP2 using nuclear extracts (E) along with expression levels of proteins in whole cell lysates (WCL), cytoplasmic (Cyto), and nuclear fractions (Nuc) (F). (G) Re‐ChIP analysis showing the occupancy of CtBP2/KLF4 complex on the human *NPY* promoter in HEK293 cells (*n* = 4). (H, I) Cell lysates from HEK293 cells expressing FLAG‐CtBP2 and/or HA‐KLF4 were incubated with increasing concentrations of NADH (0, 0.5, 5 μM, H) or oleoyl‐CoA (0, 5, 50, 500 μM, I). The bands for CtBP2 were indicated with black arrows to distinguish them from non‐specific bands. The data are expressed as the mean ± SEM with individual data points. **p < 0.01 and ***p < 0.001, as determined by Student's *t*‐test, or the two‐way ANOVA followed by Tukey's multiple comparisons test for multiple comparisons.

After observing that CtBP2 functions as a transcriptional repressor preferentially for orexigenic genes, we next surveyed the consensus motifs of CtBP2 binding sites from the ChIP‐seq data and compared those motifs against databases of known motifs of transcription factors [28] (Figure 2A). Since CtBP2 lacks DNA‐binding capabilities and relies on transcription factors to access genomic regions, we cataloged potential transcription factors that could function as effectors of CtBP2. This list included Krüppel‐like transcription factor 4 (KLF4), a known effector of CtBP2 [38], validating our analysis. This finding was also of great interest since KLF4 was reported to promote orexigenic neuropeptide expression [39, 40, 41]. Indeed, exogenous expression of KLF4 increased promoter activity of the *Npy* gene, which was repressed by exogenous expression of CtBP2, indicating that CtBP2 represses the expression of orexigenic genes through the interaction with KLF4 (Figure 2B). Conversely, knockdown of KLF4 abolished the suppressive effect of CtBP2 on the *Npy* promoter activity, suggesting that this effect is largely dependent on KLF4 (Figure 2C). The physical association between CtBP2 and KLF4 was further confirmed by our co‐immunoprecipitation experiment (Figure 2D). To determine whether this interaction occurs in the nucleus, a co‐immunoprecipitation experiment was performed using nuclear extracts, which demonstrated the interaction between CtBP2 and KLF4 in the nuclear fraction (Figure 2E,F). Furthermore, re‐ChIP analysis showed the occupancy of this transcriptional complex at the human *NPY* gene promoter (Figure 2G).

We further investigated whether this transcriptional complex responds to metabolic alterations. Increasing concentrations of NADH promoted the CtBP2/KLF4 interaction (Figure 2H), which was markedly attenuated by fatty acyl‐CoAs (Figure 2I). These results indicate that the CtBP2/KLF4 complex is dynamically regulated by distinct metabolic cues. These findings indicate that CtBP2 serves as a corepressor of orexigenic neuropeptide expression while integrating metabolic cues in this tissue.

### 3.2 Metabolic Abnormalities in Obesity Inactivate CtBP2, Resulting in Orexigenic Gene Activation

**FIGURE 3:**
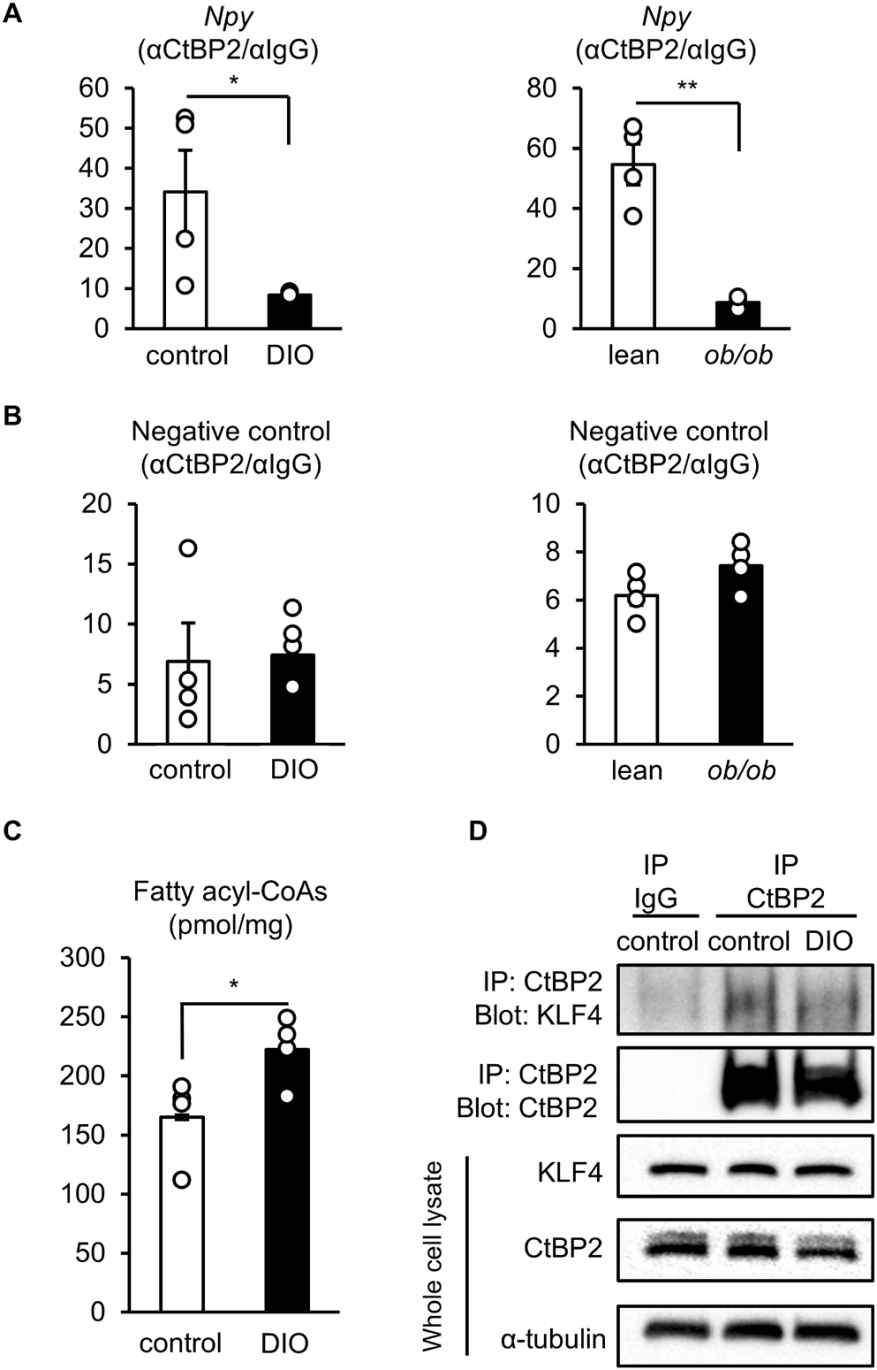
CtBP2 is inactivated in hypothalamus of obese mice. (A, B) Hypothalamic tissues were collected from genetically obese mice (*ob/ob*, 8 weeks of age) or diet‐induced obese mice (DIO, fed a high‐fat diet for 14 weeks) along with their control mice under ad libitum feeding conditions (*n* = 4). CtBP2 recruitment to the promoter regions of the *Npy* gene was assessed by ChIP‐qPCR. (C) Fatty‐acyl‐CoA levels in hypothalamic tissues from DIO mice and their controls (*n* = 4). (D) CtBP2/KLF4 complex was detected by co‐immunoprecipitation in mouse models of DIO mice and their controls (samples from three mice were pooled for each group due to the limited sample size). The data are expressed as the mean ± SEM with individual data points. *p < 0.05 and **p < 0.01, as determined by Student's *t*‐test.

We have previously reported the pivotal contributions of CtBP2 inactivation in obesity to disease development in liver and pancreatic β‐cells, with distinct modes of inactivation: allosteric inactivation induced by fatty acyl‐CoAs in the liver and CtBP2 protein degradation in pancreatic β‐cells [28, 30, 32]. Therefore, we next assessed the status of CtBP2 in the metabolic milieu in obesity in hypothalamic tissues. We found that obesity did not alter CtBP2 protein expression per se (Figure 1A), nor did it influence CtBP2 mRNA expression levels (Figure S3A). On the other hand, CtBP2 recruitment to the promoter region of the *Npy* (Figure 3A) was reduced whereas recruitment to a control region was unchanged (Figure 3B), suggesting allosteric inactivation of CtBP2 in obesity. Indeed, fatty acyl‐CoA content was increased in hypothalamic tissues of obese mice (Figure 3C). We also tried to measure the lactate/pyruvate ratio which is in equilibrium with the free NADH/NAD^+^ ratio [28, 42] and found that pyruvate concentrations were below our detection limit due to the limited sample size. Consistent with the increased fatty acyl‐CoAs, we observed dissociation of CtBP2 from KLF4 in the hypothalamic tissues of obese mice (Figure 3D). These data indicate that obesity‐induced metabolic alterations underlie the inactivation of CtBP2, which could allow KLF4 to activate orexigenic gene expression.

### 3.3 The Loss of CtBP2 in Both AgRP Neurons and POMC Neurons Leads to Increased Feeding Behavior

These data indicate that CtBP2 is allosterically inactivated in the hypothalamic tissues under obese conditions. Given that CtBP2 represses orexigenic gene expression, it may serve as a molecular link between obesity and unsuppressed overeating. To further test this hypothesis, we generated two mouse models lacking CtBP2 specifically in orexigenic AgRP neurons (AgRP neuron‐specific CtBP2 knockout, ACKO) and anorexigenic POMC neurons (POMC neuron‐specific CtBP2 knockout, PCKO) by crossing CtBP2 flox mice [28] with AgRP‐Cre transgenic and POMC‐Cre transgenic mice, respectively. The targeted deletion in each neuron was validated by immunofluorescent staining (Figure S3B). Because CtBP2 becomes dysfunctional under high‐fat diet‐induced obesity, we evaluated these mice under standard chow conditions rather than in a high‐fat diet‐induced obesity model.

**FIGURE 4:**
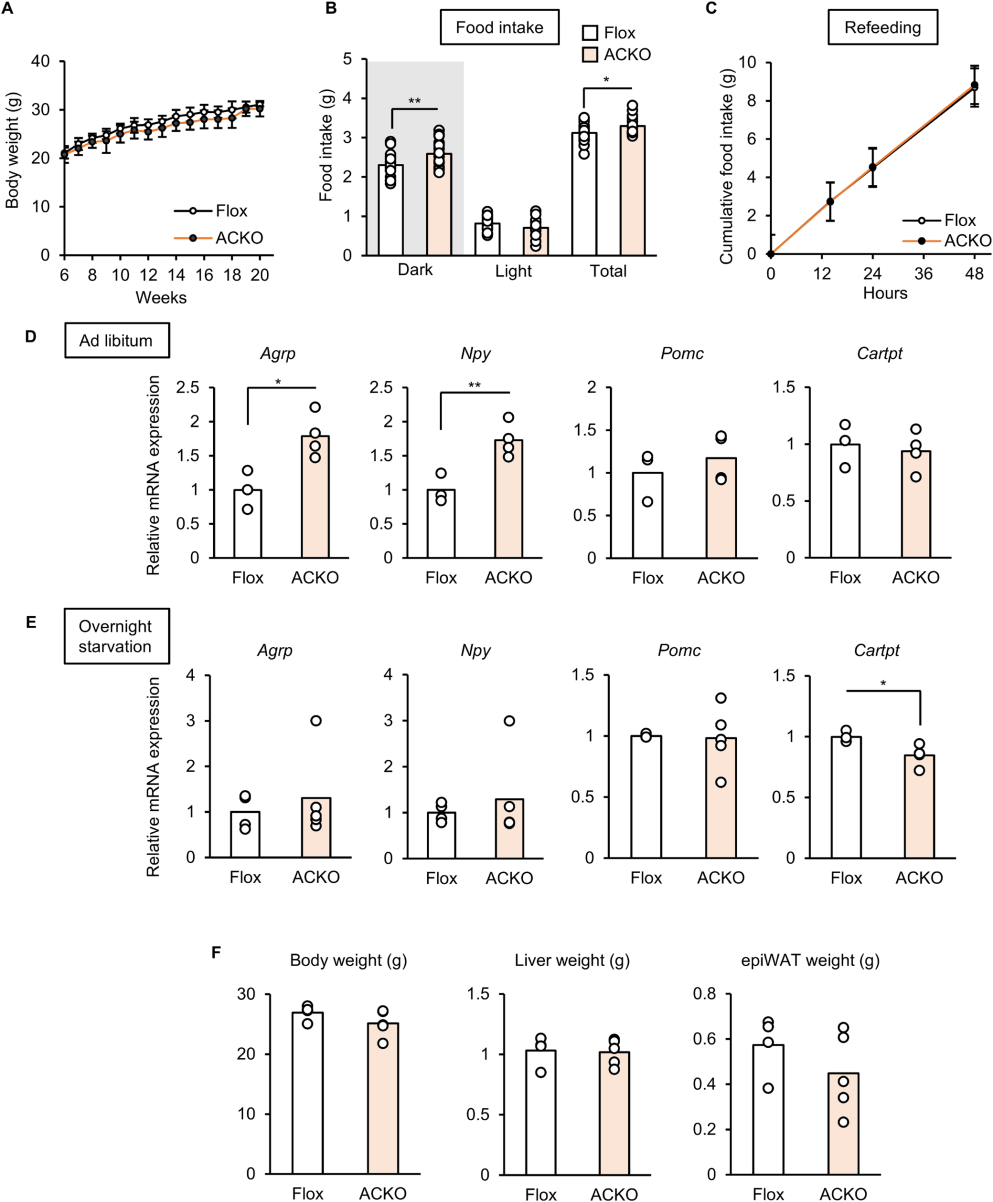
Effects of CtBP2 deficiency on AgRP neurons in vivo. (A) Growth curve of ACKO mice and their controls (Flox) (Flox: *N* = 10; ACKO: *N* = 11). (B) Twenty‐four‐h food intake (Flox: *N* = 21; ACKO: *N* = 16). (C) Food intake measured at 0, 14, 24, and 48 h during the refeeding period after overnight fasting (Flox: *N* = 11; ACKO: *N* = 8). (D, E) Orexigenic and anorexigenic neuropeptide gene expression in the hypothalamus under ad libitum conditions (Flox: *N* = 3; ACKO: N = 4; D) or overnight fasting (Flox: *N* = 4; ACKO: *N* = 5; E) in ACKO mice and their controls. (F) Body composition (Flox: *N* = 4; ACKO: *N* = 5). The data are expressed as the mean ± SEM with individual data points. *p < 0.05 and **p < 0.01, as determined by Student's *t*‐test.

In the ACKO mice maintained on a chow diet, there was no discernible difference observed in body weight gain. However, they exhibited increased cumulative daily food intake compared with their controls, which was primarily attributed to nocturnal food intake (Figure 4, A‐B). We evaluated their refeeding responses to food deprivation, which were not influenced by the genotype (Figure 4C). We examined the gene expression profile in hypothalamic tissues under two different feeding conditions: ad libitum feeding (Figure 4D) and overnight fasting (Figure 4E). As expected, we observed increased *Agrp* and *Npy* expression in the hypothalamic tissues of ACKO mice under the ad libitum feeding condition (Figure 4D), whereas this difference was not observed in the overnight fasted condition in which our feeding system exhibits a concerted effort to minimize the effects of repressors on orexigenic factors, including CtBP2 (Figure 4E). Interestingly, a body composition survey revealed a trend toward decreased epididymal adipose tissue weight in ACKO mice, although body weight and tissue weight in other tissues were unchanged (Figure 4F). In line with the body composition data, the energy expenditure of the ACKO mice tended to increase, which may also explain the maintenance of their body weight in the presence of increased food intake (Figure S4).

**FIGURE 5:**
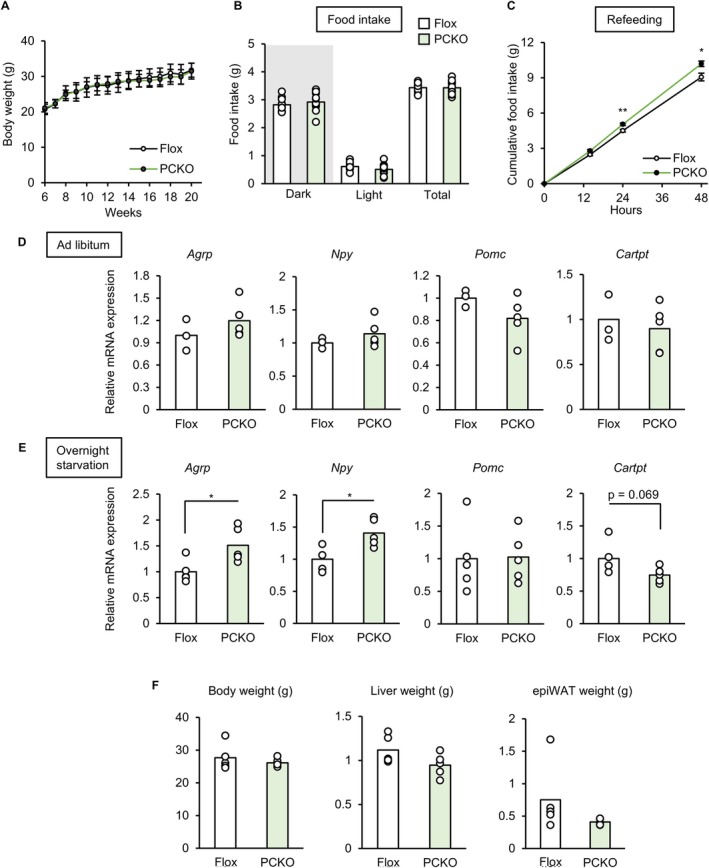
Effects of CtBP2 deficiency on POMC neurons in vivo. (A) Growth curve of the PCKO mice and their controls (Flox) (Flox: *N* = 10; PCKO: *N* = 11). (B) Twenty‐four‐h food intake (Flox: *N* = 10; PCKO: *N* = 11). (C) Food intake measured at 0, 14, 24, and 48 h during the refeeding period after overnight fasting (Flox: *N* = 7; PCKO: N = 11). (D, E) Orexigenic and anorexigenic neuropeptide gene expression in the hypothalamus under ad libitum conditions (Flox: *N* = 3; PCKO: *N* = 4; D) or overnight fasting (*n* = 5; E) in PCKO mice and their controls (*n* = 5). (F) Body composition (Flox: *N* = 4; PCKO: *N* = 5). The data are expressed as the mean ± SEM with individual data points. *p < 0.05 and **p < 0.01, as determined by Student's *t*‐test.

We next examined the PCKO mice. Similarly to the ACKO mice, there was no difference in body weight gain (Figure 5A). In contrast to the ACKO mice, the PCKO mice exhibited increased refeeding responses to 12‐h food deprivation, whereas the cumulative daily food intake under ad libitum feeding conditions was unchanged (Figure 5B,C). The levels of orexigenic gene expression were also increased in the PCKO mice (Figure 5D,E), although the difference was more evident in the overnight fasting condition (Figure 5E). Collectively, these findings suggest that, under fasting conditions, POMC neurons normally restrain orexigenic gene expression in AgRP neurons and that this regulatory mechanism is disrupted by the loss of CtBP2 in POMC neurons. Because orexigenic neuropeptides such as NPY are not expressed in POMC neurons, these results further suggest that CtBP2 in POMC neurons regulates AgRP neuronal activity through an indirect, non‐cell‐autonomous mechanism. PCKO mice also showed a trend toward decreased epididymal adipose tissue weight and increased energy expenditure, which may explain the dissociation between body weight gain and food intake (Figure 5F and Figure S5).

**FIGURE 6:**
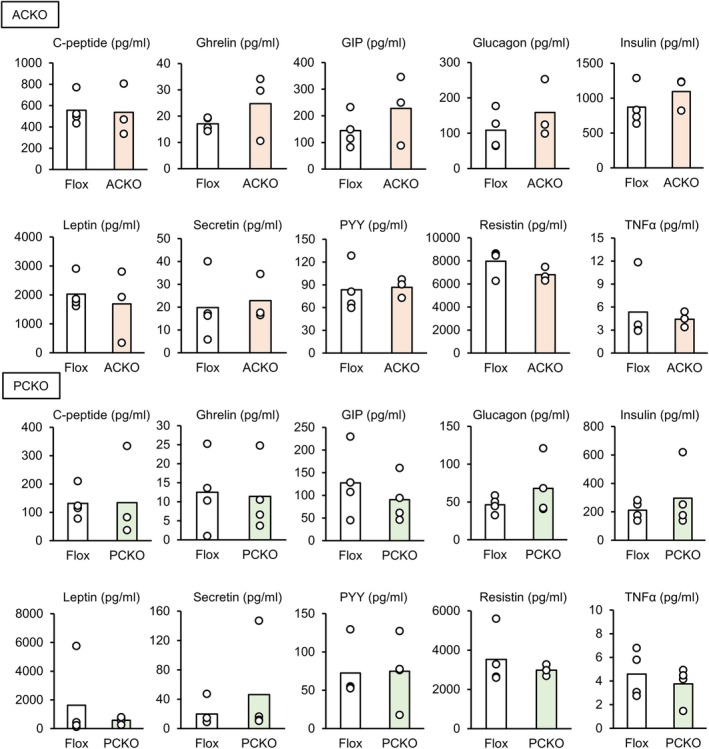
Measurement of peripheral endocrine hormones. A series of peripheral hormones were measured in the mouse models. Upper panel: ACKO mice and their controls (Flox: *N* = 4; ACKO: *N* = 3; ad libitum condition). Lower panel: PCKO mice and their controls (*n* = 4; overnight fasting condition). The ad libitum condition refers to measurements taken 7 h after the onset of the light cycle, while the overnight fasting condition refers to measurements taken at the same time point (i.e., 7 h after light onset) following a 16‐h fast initiated at the onset of the dark cycle. The data are expressed as the mean with individual data points.

Although the genetic deletion of CtBP2 increased food intake, the body weight gain in these mice remained unchanged. To investigate potential compensatory hormonal responses, we measured a series of peripheral hormones. Specifically, we utilized a multiplexed analysis encompassing leptin, ghrelin, gastric inhibitory peptide (GIP), and others. However, we did not identify any hormones responsible for this discrepancy or indirectly influencing their feeding behavior, at least within this set of hormones (Figure 6). These data indicate that obesity‐induced inactivation of CtBP2 in the hypothalamus drives orexigenic gene expression within the tissue. Whereas loss of CtBP2 in AgRP neurons directly promotes orexigenic gene expression, loss of CtBP2 in POMC neurons exerts an indirect, non‐cell‐autonomous effect on orexigenic gene expression.

### 3.4 The Transcriptome Profiles Resulting From CtBP2 Deficiency in AgRP Neurons Versus Those in POMC Neurons Exhibit Distinct Patterns

To further explore the roles of CtBP2 in transcriptional regulation within hypothalamic tissues, we utilized RNA‐seq analyses to comprehensively examine the transcriptome profiles. We aimed to determine how the loss of function of CtBP2 in a specific neuronal population (namely, AgRP neurons or POMC neurons) influences the hypothalamic transcriptional network as a whole. To this end, we conducted bulk RNA‐seq analyses on hypothalamic tissues derived from these two mouse models.

**FIGURE 7:**
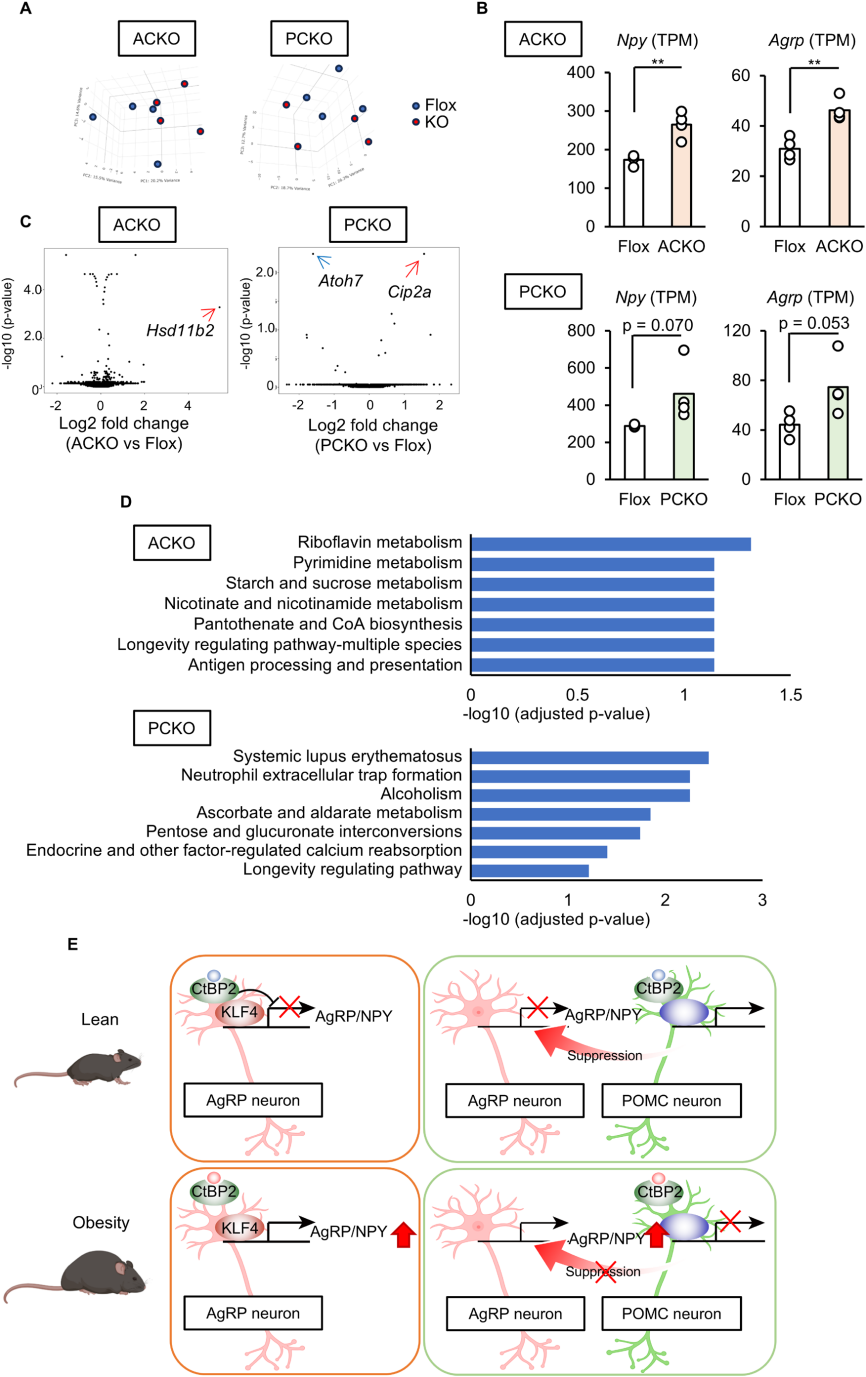
Transcriptome analyses of hypothalamic tissues from ACKO and PCKO mice. Samples were collected from ACKO mice that had access to food ad libitum and from PCKO mice that had been subjected to overnight fasting. (A) Three‐dimensional PCA. (B) Gene expression based on transcript per million (TPM) values (*n* = 4). (C) Volcano plots of differentially expressed genes (FDR < 0.25, fold change > 1.5). (D) KEGG pathway analyses. (E) Schematic representation of our proposed model. In AgRP neurons, CtBP2 directly represses the expression of orexigenic genes by targeting effector transcription factor(s). In POMC neurons, CtBP2‐mediated transcriptional alterations suppress POMC neuron activity to inhibit orexigenic gene expression in AgRP neurons. Illustrations were created with BioRender.com. The data are expressed as the mean with individual data points. **p < 0.01, as determined by Student's *t*‐test.

Principal component analysis (PCA) revealed a discernible yet modest separation between CtBP2‐knockout and control cells in both models (Figure 7A). We were able to replicate our earlier qPCR‐based targeted measurement, which revealed increased expression levels of *Agrp* and *Npy* in both knockout models (Figure 7B). Despite the shared regulatory roles in *Agrp* and *Npy* gene expression in these two different models, the analysis of differentially expressed genes (DEGs, false discovery rate (FDR) = 0.25, fold change > 1.5) revealed distinct genome‐wide alterations between these models, thus highlighting the unique roles of CtBP2 in these two neuronal populations. For instance, 11β‐hydroxysteroid dehydrogenase 2 (11β‐HSD2, *Hsd11b2*), an enzyme coupled with NADH/NAD^+^ conversion [43], was highly induced in the ACKO hypothalamus, whereas this induction was absent in the PCKO dataset (Figure 7C). Conversely, atonal homolog 7 (*Atoh7*), a transcription factor reported to be involved in neurogenesis [44], was transcriptionally suppressed, and *Cip2a*, a protein phosphatase 2A (PP2A) inhibitor reported to significantly contribute to the pathogenesis of Alzheimer's disease [45], was induced solely in PCKO hypothalamic tissues (Figure 7C). Indeed, we observed the recruitment of CtBP2 to the promoter regions of these genes, indicating direct transcriptional regulation by CtBP2 (Figure S6A,B). The results of the KEGG pathway enrichment analysis of the ACKO dataset identified riboflavin metabolism and pyrimidine metabolism, suggesting profound influences on metabolic pathways (Figure 7D). In contrast, in the PCKO dataset, systemic lupus erythematosus and neutrophil extracellular trap formation were detected, indicating potential alterations in inflammatory processes (Figure 7D). Interestingly both lists included the longevity‐regulating pathway, although it was not the top‐ranked pathway (Figure 7D). These results collectively support our model: CtBP2 directly suppresses orexigenic neuropeptide expression, which is dysregulated in obesity in AgRP neurons. On the other hand, loss of CtBP2 in POMC neurons also increases orexigenic neuropeptide expression, which should be induced in AgRP neurons, suggesting the existence of a mechanism by which changes induced in POMC neurons indirectly alter the expression of orexigenic neuropeptides in AgRP neurons. To gain further insights into this possible interneuronal regulation, we surveyed these datasets by focusing on genes exhibiting reliably high expression levels. Early growth response 1 (*Egr1*) emerged as a direct target of CtBP2 in our ChIP‐seq analysis (Figure 2A and Figure S6C) and was highly expressed in hypothalamic tissues based on TPM values (Figure S6D). Intriguingly, *Egr1* transcription was selectively suppressed in the PCKO mice (Figure S6D). Notably, EGR1 plays a critical role in synapse formation [46]. Although the expression levels of other *Egr* family genes were relatively modest, a similar trend was observed (Figure S6D). Despite potential redundancies, the lack of CtBP2 in POMC neurons may perturb formation of neural networks that propagate inhibitory signals to AgRP neurons through decreased *Egr1* expression (Figure 7E).

Collectively, the data obtained from two distinct mouse lines and cultured cells corroborate our model in which, under physiological conditions, CtBP2 restrains orexigenic gene expression through distinct neuronal mechanisms: cell‐autonomously by repressing KLF4 in AgRP neurons and indirectly through a non‐cell‐autonomous, albeit as yet elusive, pathway in POMC neurons. Obesity disrupts this CtBP2‐dependent homeostatic mechanism, thereby contributing to the excessive food intake characteristic of obesity (Figure 7E).

## 4 Discussion

Several vital components, such as body temperature and water balance, are maintained within a narrow range, and similarly, we have a set point for body weight. The act of seeking food is one of the behaviors that serves to protect against weight deviations, and dysregulation of this adaptive response can lead to obesity. In this study, we demonstrated that the metabolite sensor CtBP2 could serve as a molecular link between obesity and maladaptive feeding behavior. CtBP2 is inactivated in response to the abnormal metabolic milieu induced by obesity, leading to an orexigenic expression profile in the hypothalamus.

Although decreased orexigenic gene expression and reduced food intake are sometimes observed in high‐fat diet‐induced obesity models [47], these phenotypes likely reflect the net effect of multiple biological processes that either promote or suppress feeding. Among these, processes that promote food intake remain important for obesity development, and inactivation of CtBP2 contributes to this orexigenic drive.

CtBP2 is metabolically activated by NADH/NAD^+^ with preference for NADH and inactivated by fatty acyl‐CoAs. Indeed, obesity increases the levels of acyl‐CoAs and their derivatives in the brain and other tissues [28, 48, 49, 50, 51], which was also the case in hypothalamic tissues as shown in this study (Figure 3C). In addition, the structural commonality of NAD(H) and acyl‐CoAs is the adenosine structure, and it remains possible that other metabolites sharing this structure may regulate CtBP2 in hypothalamic tissues. From another perspective focusing on NADH, overeating increases glycolytic flux, resulting in NADH production. Given that NADH activates CtBP2 to suppress feeding behavior, CtBP2 plays a safeguarding role in maintaining the homeostatic control of feeding behavior by limiting excessive food intake.

It has been reported that excess NADH induces reductive stress which is detrimental to metabolic homeostasis [52], whereas NADH‐mediated CtBP2 activation provides diverse metabolic benefits. We reconciled these seemingly contradictory observations using the concept of hormesis [33]: high‐level exposure to a stressor is harmful, whereas low‐level exposure elicits beneficial adaptive responses by increasing resilience [53], resulting in improved organismal fitness [54]. We reported that one of the key roles of CtBP2 is bolstering metabolic resilience [33]. While excess NADH at pathologically high concentrations is toxic to living organisms, CtBP2 detects early warning signs of NADH surplus and protects against the detrimental effects by increasing resilience. This hormetic framework is applicable to our body weight regulation: body weight is maintained within a narrow range, and deviations from this set point trigger several adaptive responses. Modest overconsumption elicits adaptive responses that prevent sustained weight gain, whereas excessive overnutrition overwhelms these adaptive mechanisms and leads to obesity. CtBP2 is in part responsible for the adaptation, and excessive overnutrition metabolically inactivates CtBP2, resulting in impairment of this adaptive system. Metabolism would be tightly linked to these adaptive responses, and investigations focusing on CtBP2 may offer clues to understanding these metabolic adaptations in a wide range of biological processes.

We observed orexigenic gene expression under ad libitum conditions in ACKO mice and during fasting conditions in PCKO mice. In AgRP neurons, orexigenic neuropeptide expression is typically suppressed when food is abundant, partly due to CtBP2‐mediated transcriptional repression. We identified KLF4 as the target of CtBP2 in this context, although functional redundancy with other KLF family members cannot be excluded. POMC neuron activities to compete with AgRP neurons should be minimized in starved conditions, and this suppression of POMC neuron activity would be in part regulated by CtBP2‐mediated transcriptional repression, although the direct targets of CtBP2 in this process remain unknown. In line with our observations, KLF4 was reported to function specifically in AgRP neurons [40]. These findings suggest that a lack of CtBP2 in POMC neurons may indirectly regulate orexigenic gene expression in AgRP neurons (Figure 7E).

Although the observed increases in food intake in the ACKO and PCKO mice were relatively modest, these outcomes align with those of many loss‐of‐function studies. Even the absence of AgRP and/or NPY only marginally increases food intake solely under certain conditions, if at all [5, 6, 7, 8, 9, 10]. Similarly, the lack of insulin receptors in the central nervous system marginally increases food intake exclusively in female mice [55]. A notable exception is leptin signaling deficiency: the absence of leptin itself and the leptin receptor results in a massive increase in food consumption [2]. This redundancy reflects the importance and indispensable nature of feeding behavior for the survival of living systems. Concurrent loss of CtBP2 in AgRP and POMC neurons might yield a more robust increase in food intake. Moreover, considering the broad expression of CtBP2 across nearly all cell types, its role in other brain feeding centers such as the nucleus accumbens, ventral tegmental area, and nucleus tractus solitarius should be considered. Compensatory changes induced by the loss of CtBP2 may also underlie the phenotypic manifestations. The reciprocal interconversion of glucocorticoids by 11β‐HSD1 and 11β‐HSD2 regulates local glucocorticoid activity in the central feeding center, thereby influencing the rhythmic regulation of AgRP neurons [56]. The increased expression of 11β‐HSD2 in ACKO may reflect a compensatory response. From a systemic perspective, the increased energy expenditure observed in ACKO and PCKO mice may be a compensatory response to maintain body weight within a narrow range. In ACKO and PCKO mice, the adaptive responses to maintain body weight are intact except in the hypothalamic feeding center. Although we were not able to identify the humoral factors responsible for this effect, other systems such as afferent projections to brown adipose tissue warrant consideration [57]. Considering the potential body‐wide contributions of CtBP2 inactivation to obesity [28, 29, 30, 32], simultaneous inactivation of CtBP2 in other tissues may attenuate these compensatory responses, which deserves further scrutiny.

A limitation of this study involves the lack of approaches specific to neuronal subpopulations. For instance, the separate characterization of the transcriptomes of POMC neurons and AgRP neurons in the PCKO mice could provide deeper insights into the mechanisms underlying the indirect suppressive effects of POMC neurons on AgRP neurons. Because our RNA‐seq analyses assessed whole hypothalamic tissue rather than the specific neuronal population lacking CtBP2, we were unable to apply highly stringent statistical criteria, such as a low FDR threshold, without compromising the sensitivity for detecting differentially expressed genes. Our ChIP‐seq was also not performed at single‐cell resolution. Many state‐of‐the‐art techniques such as transcriptome analyses at the single‐cell level, optogenetics, and spatial transcriptomics have been used to further address this question. Future studies utilizing these techniques would convincingly clarify the questions that remain unclear. In addition, the energy expenditure in ACKO and PCKO may represent adaptive responses to increased food intake; however, the observations in this study might be coincidental, and longitudinal studies are required to establish causal relationships. There might also be other compensatory mechanisms behind this discrepancy between food intake and weight gain.

Despite the limitations, we believe our findings are notable in that they expand upon our previous work establishing the potential role of CtBP2 inactivation in the pathogenesis of obesity [32]. The global obesity pandemic is a major health threat, necessitating the development of therapeutic approaches for obesity and its associated diseases. Our current study provides a basis for better understanding the pathogenesis of obesity and for developing promising pharmacological approaches. We have demonstrated that functional defects in CtBP2 in obesity lead to hallmark characteristics of the disease in liver and pancreatic β‐cells, and that the activation of CtBP2 can mitigate the metabolic deteriorations associated with obesity [28, 29, 30, 32]. Our present study suggests that CtBP2 activation in the feeding center may reprogram the abnormal body weight set point in obesity. Although we could not examine the effects of restoration of CtBP2 activity in hypothalamic tissues of obese mice due to several technical challenges in this study, development of pharmacological approaches in the future may solve this uncertainty. To consider potential clinical applications, validation using human samples would greatly enhance feasibility although it remains highly challenging. However, given that CtBP2 shares 99% homology between humans and mice, it is plausible that the phenomena observed in mice may also be applicable to humans. Therefore, the activation of CtBP2 could be an attractive strategy for targeting multiple tissues and cells. CtBP2 regulates transcription through epigenetic modulation, playing a critical role in metabolic imprinting or metabolic memory [58]. Since obesity is a chronic disease that progresses over an extended period of time, strategies targeting CtBP2 hold promise for future clinical approaches from this perspective. We also reported that activated CtBP2 is secreted via exosomes to protect against aging processes [33]. Activated CtBP2 may contribute to the well‐established pro‐longevity effects of caloric restriction [59] in part through its appetite‐suppressing actions. When integrated with our preceding observations, the findings in this study indicate that the activation of CtBP2 could be an attractive strategy for a wide range of health challenges ranging from obesity to aging by acting on multiple tissues and cells.

## Author Contributions

W.C., K.K., K.S., and M.S. performed most of the experiments. K.K. performed the Milliplex mouse metabolic hormone expanded panel measurements; TM1 (Takaaki Matsuda) performed the initial screening experiments using the mHypoE N41 cell line. D.Y., Y.K., A.N., and N.A.‐S. performed the prerequisite experiments for the final data. TM2 (Takafumi Miyamoto) and TM3 (Takashi Matsuzaka) provided critical expertise. Y.M., Y.S., Y.O., H.I., and H.S. provided substantial intellectual advice required for data collection. M.S. conceived and designed the study and wrote the manuscript with W.C.

## Funding

This work was supported by MEXT|Japan Society for the Promotion of Science (JSPS), (26K02846, 23K18270); Japan Agency for Medical Research and Development (AMED), JP18gm5910007, JP25gm6710004, JP22ek0210175; Takeda Science Foundation (TSF), NA; Ono Medical Research Foundation, NA; Manpei Suzuki Diabetes Foundation, NA; Japan Diabetes Foundation, NA; Astellas|Astellas Foundation for Research on Metabolic Disorders (AFRMD), NA; Suzuken Memorial Foundation (SMF), NA; Japan Agency for Medical Research and Development (AMED), JP256f0137008.

## Ethics Statement

The research protocol received approval from the Animal Care Committee, University of Tsukuba (approval number 25‐113), and all of the experimental procedures involving animals were performed in accordance with the committee's guidelines.

## Conflicts of Interest

The authors declare no conflicts of interest.

## Supporting information


**Figure S1:** Global mapping of CtBP2 binding sites by ChIP‐seq. (A) CtBP2 ChIP‐seq peaks at the *Cartpt* and *Pomc* gene loci. (B) CtBP2 ChIP‐seq peaks at the *Gal*, *Galr1*, *Hcrt*, *Hcrtr1*, *Hcrtr2*, *Pmch* and *Mchr1* gene loci.
**Figure S2:** CtBP2 ChIP‐seq peaks at the loci of representative genes. (A) Genes related to heterochromatin assembly. (B) Genes related to PRC1 complex formation.
**Figure S3:** Expression levels of *Ctbp2* mRNA in hypothalamic tissues, and validation of mouse models. (A) Genetically obese mice (*ob/ob*) and their controls (*n* = 4). Diet induced obese mice (DIO) and their controls (*n* = 3). (B) Representative immunofluorescence images of hypothalamic sections from Flox, ACKO and PCKO mice. Sections were stained with DAPI (blue), CtBP2 (green) and either AgRP or POMC (red), and merged images are shown. 3v, third ventricle. Scale bar = 100 μm. The yellow box indicates the selected region, which is shown at higher magnification below. White arrows indicate the presence or absence of CtBP2 in representative cells. The data are expressed as the mean with individual data points. *p < 0.05, as determined by Student's *t*‐test.
**Figure S4:** Energy expenditure in ACKO mice Oxygen and carbon dioxide production (VO_2_ and VCO_2_, respectively) were measured by indirect calorimetry in ACKO mice and their controls (*n* = 6). The respiratory quotient was determined as the ratio of VCO_2_/VO_2_. The data are expressed as the mean ± SEM.
**Figure S5:** Energy expenditure in PCKO mice Oxygen and carbon dioxide production (VO_2_ and VCO_2_, respectively) were measured by indirect calorimetry in the PCKO mice and their controls (*n* = 8). The respiratory quotient was determined as the ratio of VCO_2_/VO_2_. The data are expressed as the mean ± SEM. *p < 0.05, as determined by Student's *t*‐test.
**Figure S6:** Transcriptional landscapes of hypothalamic tissues in ACKO and PCKO A‐B. ChIP‐seq peaks at the *Hsb11b2* (A), *Atoh7* and *Cip2a* (B) gene loci. (C) ChIP‐seq peak at the *Egr1* promoter. (D) Expression levels of EGR family transcription factors. The data are expressed as the mean with individual data points.

## Data Availability

ChIP‐seq and RNA‐seq data were deposited in the Gene Expression Omnibus under accession numbers GSE266964 (ChIP‐seq), GSE266965 (RNA‐seq for ACKO), and GSE266966 (RNA‐seq for PCKO). Other data and materials used in this study are available upon reasonable request.
