# Supplementary material for: Obesity Disrupts CtBP2‐Mediated Maintenance of Transcriptional Equilibrium in Hypothalamic Feeding Circuitry
Source: FASEB J. 2026 Jul 29;40(15):e72172. doi: 10.1096/fj.202602322R (PMC13417749; doi:10.1096/fj.202602322R)

Supplemental Figure 1

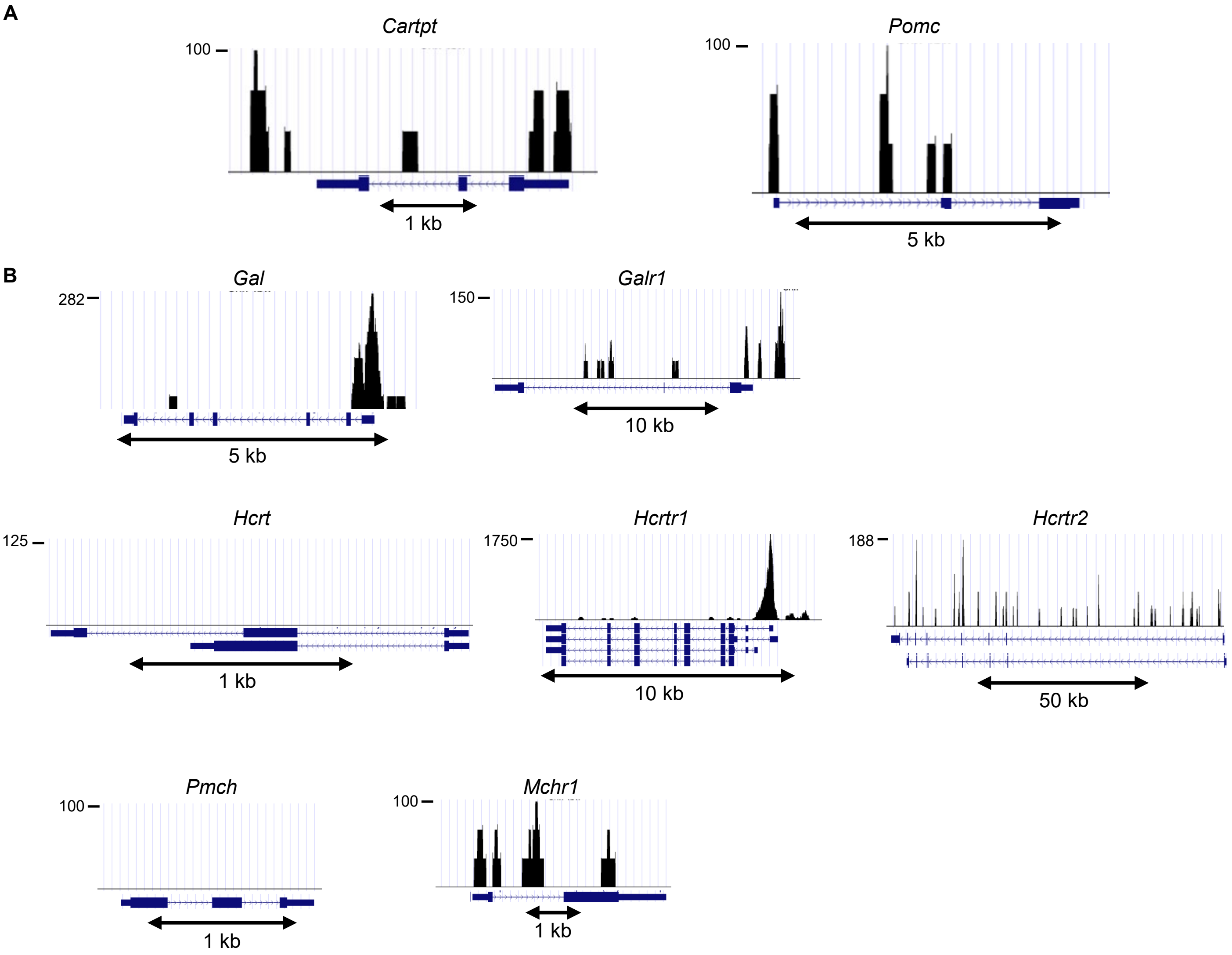

Supplemental Figure 2

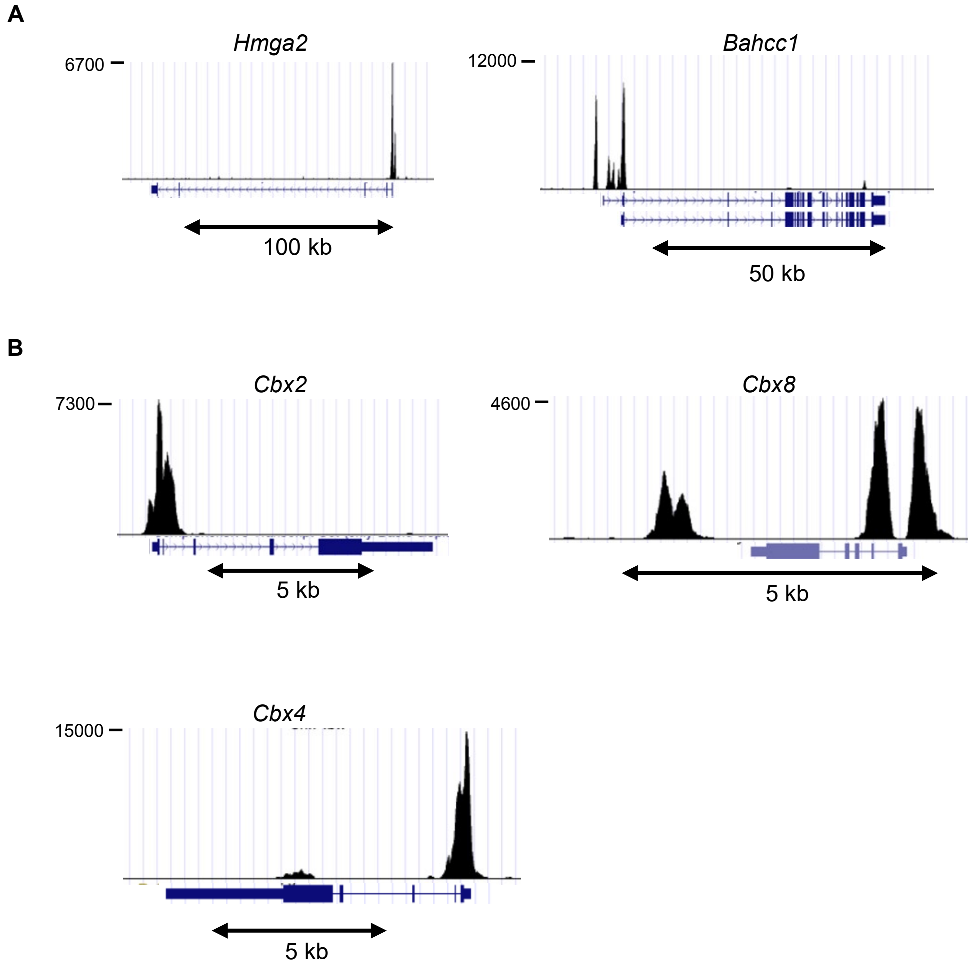

Supplemental Figure 3

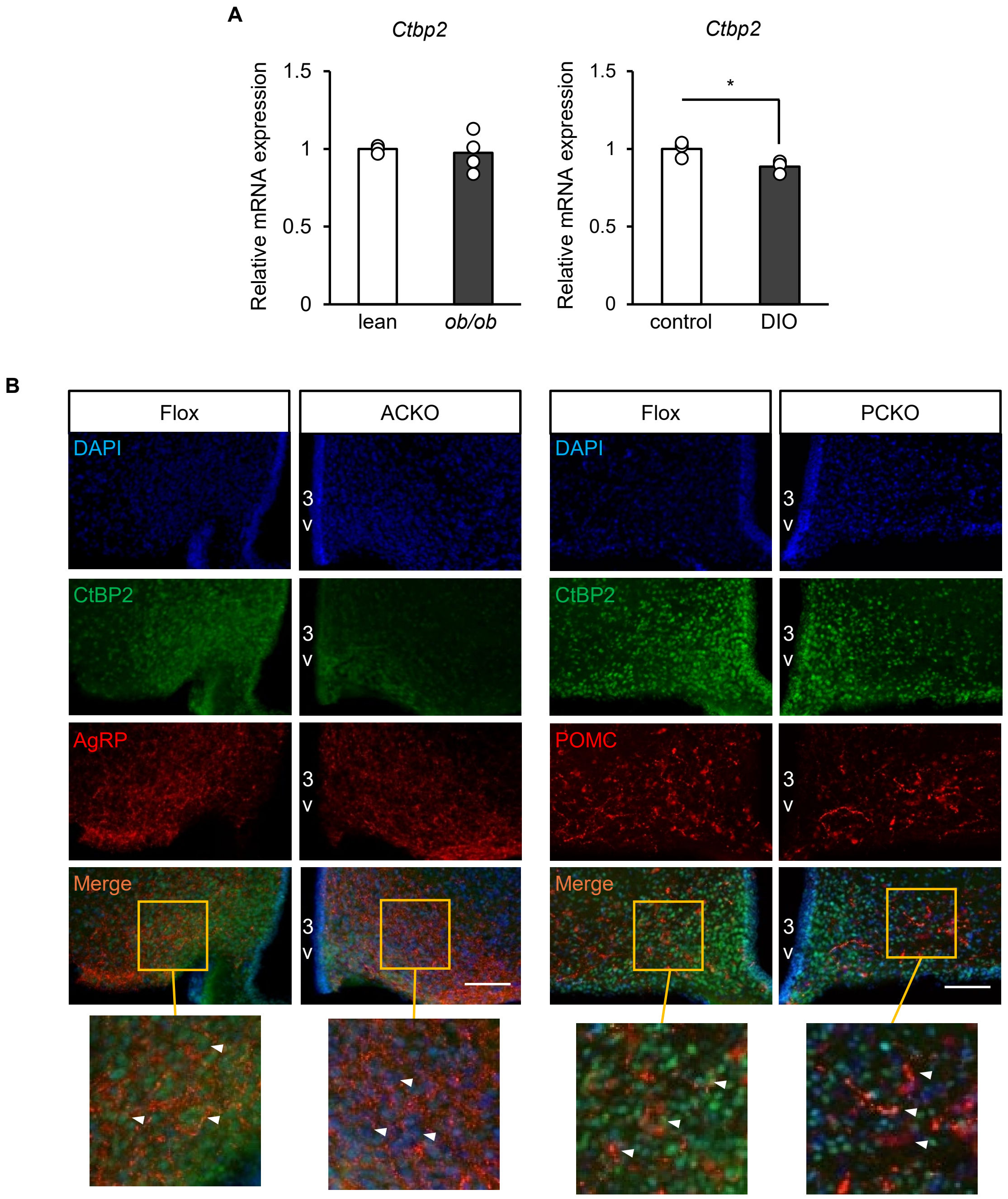

Supplemental Figure 4

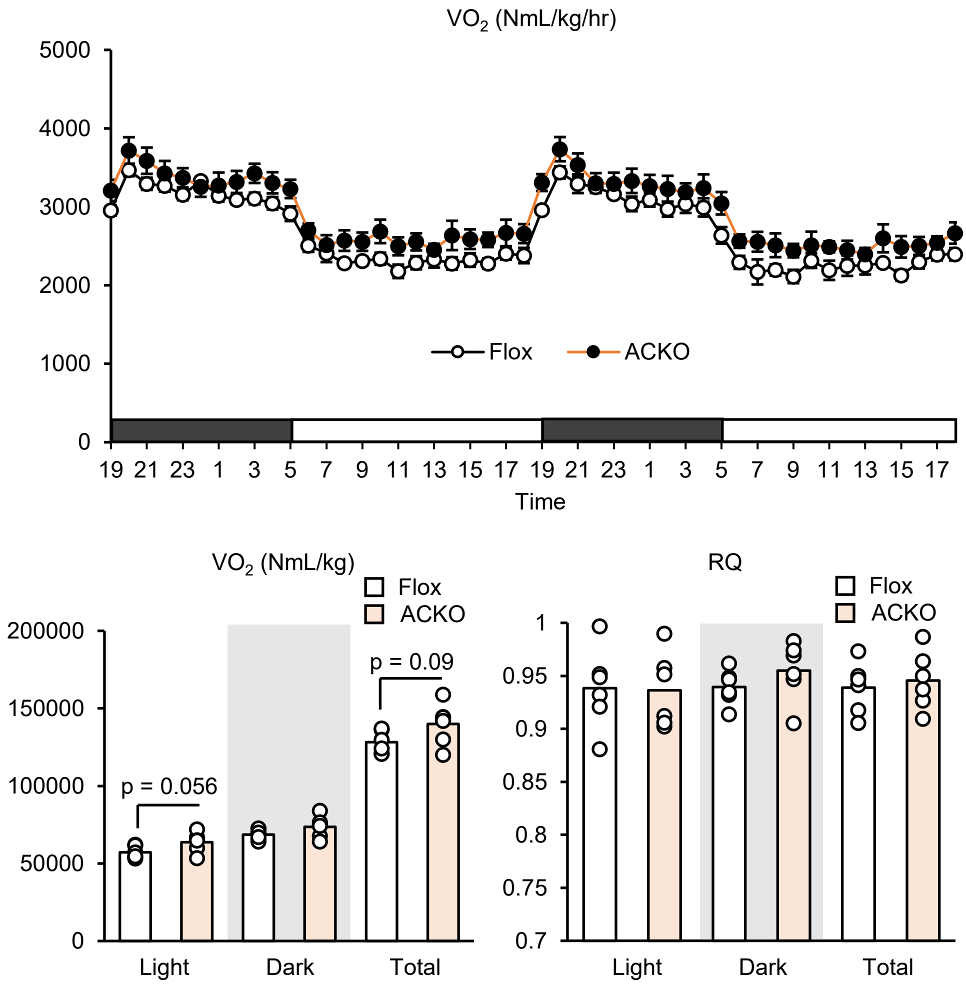

Supplemental Figure 5

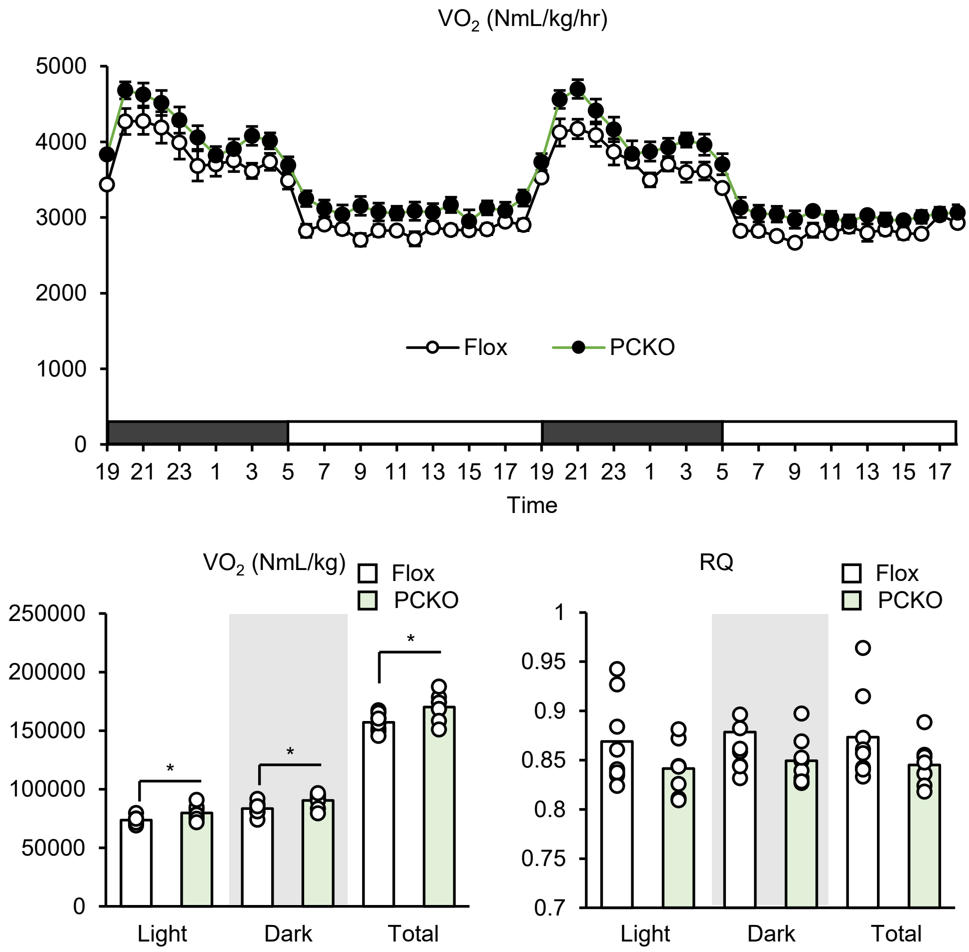

Supplemental Figure 6

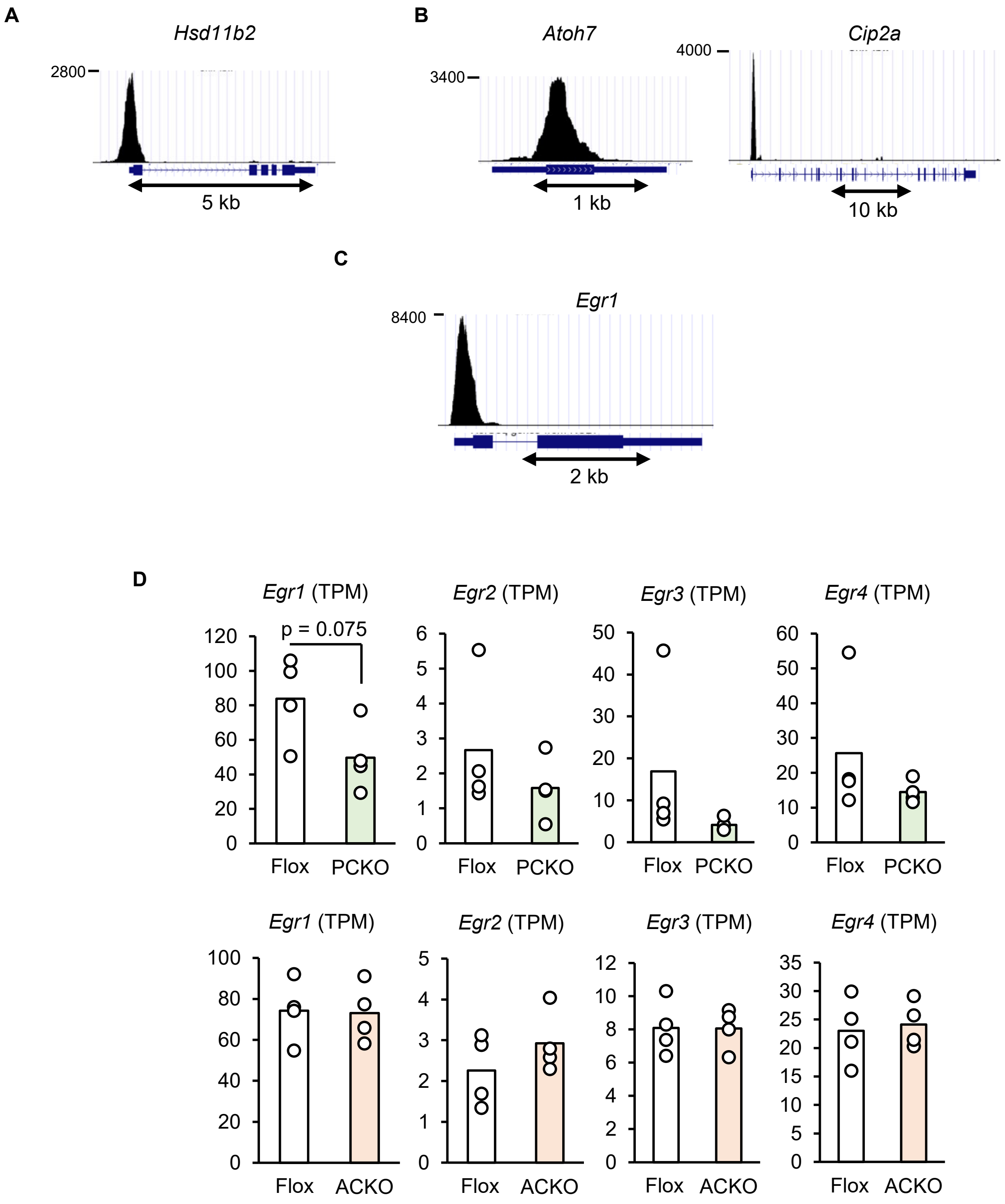

Supplement: Supplementary file 1 — Figure S1: Global mapping of CtBP2 binding sites by ChIP‐seq. (A) CtBP2 ChIP‐seq peaks at the Cartpt and Pomc gene loci. (B) CtBP2 ChIP‐seq peaks at the Gal, Galr1, Hcrt, Hcrtr1, Hcrtr2, Pmch and Mchr1 gene loci. Figure S2: CtBP2 ChIP‐seq peaks at the loci of representative genes. (A) Genes related to heterochromatin assembly. (B) Genes related to PRC1 complex formation. Figure S3: Expression levels of Ctbp2 mRNA in hypothalamic tissues, and validation of mouse models. (A) Genetically obese mice (ob/ob) and their controls (n = 4). Diet induced obese mice (DIO) and their controls (n = 3). (B) Representative immunofluorescence images of hypothalamic sections from Flox, ACKO and PCKO mice. Sections were stained with DAPI (blue), CtBP2 (green) and either AgRP or POMC (red), and merged images are shown. 3v, third ventricle. Scale bar = 100 μm. The yellow box indicates the selected region, which is shown at higher magnification below. White arrows indicate the presence or absence of CtBP2 in representative cells. The data are expressed as the mean with individual data points. *p < 0.05, as determined by Student's t‐test. Figure S4: Energy expenditure in ACKO mice Oxygen and carbon dioxide production (VO2 and VCO2, respectively) were measured by indirect calorimetry in ACKO mice and their controls (n = 6). The respiratory quotient was determined as the ratio of VCO2/VO2. The data are expressed as the mean ± SEM. Figure S5: Energy expenditure in PCKO mice Oxygen and carbon dioxide production (VO2 and VCO2, respectively) were measured by indirect calorimetry in the PCKO mice and their controls (n = 8). The respiratory quotient was determined as the ratio of VCO2/VO2. The data are expressed as the mean ± SEM. *p < 0.05, as determined by Student's t‐test. Figure S6: Transcriptional landscapes of hypothalamic tissues in ACKO and PCKO A‐B. ChIP‐seq peaks at the Hsb11b2 (A), Atoh7 and Cip2a (B) gene loci. (C) ChIP‐seq peak at the Egr1 promoter. (D) Expression levels of EGR family [file FSB2-40-e72172-s001.zip › fsb272172-sup-0002-Supinfo02.pdf]
