# Supplementary material for: Obesity Disrupts CtBP2‐Mediated Maintenance of Transcriptional Equilibrium in Hypothalamic Feeding Circuitry
Source: FASEB J. 2026 Jul 29;40(15):e72172. doi: 10.1096/fj.202602322R (PMC13417749; doi:10.1096/fj.202602322R)
Supplement: Supplementary file 1 — Figure S1: Global mapping of CtBP2 binding sites by ChIP‐seq. (A) CtBP2 ChIP‐seq peaks at the Cartpt and Pomc gene loci. (B) CtBP2 ChIP‐seq peaks at the Gal, Galr1, Hcrt, Hcrtr1, Hcrtr2, Pmch and Mchr1 gene loci. Figure S2: CtBP2 ChIP‐seq peaks at the loci of representative genes. (A) Genes related to heterochromatin assembly. (B) Genes related to PRC1 complex formation. Figure S3: Expression levels of Ctbp2 mRNA in hypothalamic tissues, and validation of mouse models. (A) Genetically obese mice (ob/ob) and their controls (n = 4). Diet induced obese mice (DIO) and their controls (n = 3). (B) Representative immunofluorescence images of hypothalamic sections from Flox, ACKO and PCKO mice. Sections were stained with DAPI (blue), CtBP2 (green) and either AgRP or POMC (red), and merged images are shown. 3v, third ventricle. Scale bar = 100 μm. The yellow box indicates the selected region, which is shown at higher magnification below. White arrows indicate the presence or absence of CtBP2 in representative cells. The data are expressed as the mean with individual data points. *p < 0.05, as determined by Student's t‐test. Figure S4: Energy expenditure in ACKO mice Oxygen and carbon dioxide production (VO2 and VCO2, respectively) were measured by indirect calorimetry in ACKO mice and their controls (n = 6). The respiratory quotient was determined as the ratio of VCO2/VO2. The data are expressed as the mean ± SEM. Figure S5: Energy expenditure in PCKO mice Oxygen and carbon dioxide production (VO2 and VCO2, respectively) were measured by indirect calorimetry in the PCKO mice and their controls (n = 8). The respiratory quotient was determined as the ratio of VCO2/VO2. The data are expressed as the mean ± SEM. *p < 0.05, as determined by Student's t‐test. Figure S6: Transcriptional landscapes of hypothalamic tissues in ACKO and PCKO A‐B. ChIP‐seq peaks at the Hsb11b2 (A), Atoh7 and Cip2a (B) gene loci. (C) ChIP‐seq peak at the Egr1 promoter. (D) Expression levels of EGR family [file FSB2-40-e72172-s001.zip › fsb272172-sup-0001-Supinfo01.docx]

**Supplementary Information**

**Obesity disrupts CtBP2-mediated maintenance of transcriptional equilibrium in hypothalamic feeding circuitry**

Authors: Wanpei Chen^1^, Kenta Kainoh^1^, Kenji Saito^1^, Takaaki Matsuda^1^, Daichi Yamazaki^1^, Yuto Kobari^1^, Ayumi Nakata^1^, Nao Aono-Soma^1^, Takafumi Miyamoto^1,2^, Yuki Murayama^1^, Yoko Sugano^1^, Yoshinori Osaki^1^, Hitoshi Iwasaki^1^, Takashi Matsuzaka^1,3^, Hitoshi Shimano^1^, Motohiro Sekiya^1,2,#^

**Supplemental figure legends**

Supplemental Figure 1. Global mapping of CtBP2 binding sites by ChIP-seq.

A. CtBP2 ChIP-seq peaks at the *Cartpt* and *Pomc* gene loci.

B. CtBP2 ChIP-seq peaks at the *Gal*, *Galr1*, *Hcrt*, *Hcrtr1*, *Hcrtr2*, *Pmch* and *Mchr1* gene loci.

Supplemental Figure 2. CtBP2 ChIP-seq peaks at the loci of representative genes.

A. Genes related to heterochromatin assembly.

B. Genes related to PRC1 complex formation.

Supplemental Figure 3. Expression levels of *Ctbp2* mRNA in hypothalamic tissues, and validation of mouse models.

A. Genetically obese mice (*ob/ob*) and their controls (n = 4). Diet induced obese mice (DIO) and their controls (n = 3).

B. Representative immunofluorescence images of hypothalamic sections from Flox, ACKO and PCKO mice. Sections were stained with DAPI (blue), CtBP2 (green) and either AgRP or POMC (red), and merged images are shown. 3v, third ventricle. Scale bar = 100 μm. The yellow box indicates the selected region, which is shown at higher magnification below. White arrows indicate the presence or absence of CtBP2 in representative cells. The data are expressed as the mean with individual data points. *p < 0.05, as determined by Student’s t-test.

Supplemental Figure 4. Energy expenditure in ACKO mice

Oxygen and carbon dioxide production (VO_2_ and VCO_2_, respectively) were measured by indirect calorimetry in ACKO mice and their controls (n = 6). The respiratory quotient was determined as the ratio of VCO_2_/VO_2_. The data are expressed as the mean ± SEM.

Supplemental Figure 5. Energy expenditure in PCKO mice

Oxygen and carbon dioxide production (VO_2_ and VCO_2_, respectively) were measured by indirect calorimetry in the PCKO mice and their controls (n = 8). The respiratory quotient was determined as the ratio of VCO_2_/VO_2_. The data are expressed as the mean ± SEM. *p < 0.05, as determined by Student’s t-test.

Supplemental Figure 6: Transcriptional landscapes of hypothalamic tissues in ACKO and PCKO

A-B. ChIP-seq peaks at the *Hsb11b2* (A), *Atoh7* and *Cip2a* (B) gene loci.

C. ChIP-seq peak at the *Egr1* promoter.

D. Expression levels of EGR family transcription factors. The data are expressed as the mean with individual data points.
